# Performance of Large Language Models in the Cognitive Analysis of Misinformation: Evaluation Study

**DOI:** 10.2196/72524

**Published:** 2026-05-18

**Authors:** Dominika Nadia Wojtczak, Cheryl McQuire, Luisa Zuccolo, Claudia Peersman, Ryan McConville

**Affiliations:** 1 School of Computer Science University of Bristol Bristol United Kingdom; 2 Bristol Medical School University of Bristol Bristol United Kingdom; 3 Health Data Science Centre Human Technopole Milan Italy; 4 Bristol Medical School Department of Population Health Sciences University of Bristol Bristol United Kingdom; 5 School of Engineering Mathematics and Technology University of Bristol Bristol United Kingdom

**Keywords:** online misinformation, LLM, fake news, cognitive analysis, panel of annotators, ground truth, intent, reasoning, cognitive reasoning, large language model

## Abstract

**Background:**

Public discourse is significantly impacted by the rapid spread of misinformation on social media platforms. Human moderators, while capable of performing well, face many challenges due to scalability. While large language models (LLMs) show great potential across various language tasks, their capacity for cognitive and contextual analysis, in detecting and interpreting misinformation, remains less evaluated.

**Objective:**

This study evaluates the effectiveness of LLMs in detecting and interpreting misinformation compared to human annotators, focusing on tasks requiring cognitive analysis and complex judgment. Additionally, we analyze the influence of different prompt engineering strategies on model performance and discuss ethical considerations for using LLMs in content moderation systems.

**Methods:**

We evaluated 4 OpenAI models against a panel of human annotators using a subset of posts from the MuMiN dataset. Each model and human annotator responded to structured questions on misinformation, following an established cognitive framework. Both human annotators and LLMs also provided scores indicating how confident they were in their responses. Various prompting strategies were used in this research, including: 0-shot, few-shot, and chain-of-thought, with performance evaluated through precision, recall, *F*_1_-score, and accuracy. We used statistical tests, including the McNemar test, to quantitatively assess differences between LLM and human ratings of misinformation.

**Results:**

GPT-4 Turbo with chain-of-thought prompting achieved the highest performance of all LLMs for detecting misinformation, with an accuracy of 67.2% and an *F*_1_-score of 78.3%, but was outperformed by human annotators, who achieved 70.1% accuracy and an *F*_1_-score of 81%. LLMs performed well in tasks involving logical reasoning and straightforward misinformation detection, but struggled with complex judgments, including detecting sarcasm, understanding misinformation, and analyzing user intent. LLM confidence scores positively correlated with accuracy in simpler tasks (r=0.72, *P*<.01) but were less reliable in subjective and complex contextual evaluations.

**Conclusions:**

LLMs show significant potential for automating misinformation detection. Their limitations in understanding and interpreting these posts highlight the current necessity of human oversight. A hybrid framework combining LLMs for preliminary screening with human moderators for more complex evaluation presents a promising future direction. Future research could prioritize the fine-tuning of LLMs using datasets that emphasize cognitive and emotional linguistic features, alongside the development of advanced prompting techniques.

## Introduction

### Online Misinformation

Political leaflets in ancient Rome and the Great Moon Hoax of 1835 remind us that societies have long struggled with falsehoods; however, the scale, speed, and sociotechnical context of the problem have changed radically in the social media era [1].

Social media platforms have become major hubs of misinformation, significantly influencing public opinion, political discourse, and public health decisions [2,3]. Platforms such as X (formerly known as Twitter, X Corp) provide a global space for communication about current affairs, allowing users to share their perspectives widely. Still, they also enable the rapid spread of misinformation, which can distort perceptions of reality, damage trust, and contribute to polarized echo chambers leading to the viral spread of online misinformation [4,5]. Users may explore credibility on social platforms through social factors that influence their perception of misinformation, in addition to factual verification of posts [6].

The current research distinguishes misinformation (false content shared without intent to deceive), disinformation (false content shared with intent), and malinformation (genuine information taken out of context that is shared maliciously to harm individuals, groups, or society). These distinctions highlight the variety of false content, which can differ in origin, intent, and potential harm [7-9]. Nonetheless, detecting these features at scale remains challenging. The MuMiN definition of misinformation is used in this study, which classifies any verified (and mentioned) falsehood as misinformation [10]. While it remains crucial for maintaining conceptual clarity, consolidating all misleading content under a single label supports the large-scale annotation and detection efforts. We adopt MuMiN’s definition because it tries to provide a similar method to real‑world moderation pipelines, where the primary question is simply: is this claim factually wrong? And therefore, the same question is what our paper poses to both human annotators and large language models (LLMs). This methodology forms the foundation of our dataset development [11-13]. Moreover, we do not differentiate these categories in this study due to the lack of intent annotations in the MuMiN dataset. However, our cognitive framework includes perceived intent as a feature, and future research may more clearly separate these concepts.

Spreading misinformation can result in serious risks to society [14]. For example, false narratives can manipulate voter behavior [15], increase social divisions [16,17], and spread misinformation about diseases, treatments, or vaccines that can lead to dangerous actions, as seen during the COVID-19 pandemic [2] or increase social divisions as before it. The rapid spread of misinformation, which can be intensified by algorithms that promote engaging content, poses a significant challenge for both platforms and policy regulators.

Traditional methods of content moderation relying mainly on human moderators are becoming insufficient as misinformation grows in both volume and complexity. The amount of content makes it impossible for human moderators to manually evaluate every potential instance of misinformation. Furthermore, the nature of this content can also be harmful to human moderators’ well-being [18].

### About LLMs

Given these challenges, approaches involving generative artificial intelligence (AI) have been explored to address the issue of misinformation detection at scale. LLMs such as GPT-4 have gained attention for their ability to generate responses to a wide range of prompts. These models can perform various language tasks, including sentiment analysis and text summarization, which has led to their application in content moderation and misinformation detection.

Nevertheless, despite their advancements, these models have limitations. Although LLMs exhibit extensive knowledge, they lack an understanding of commonsense knowledge [19]. Therefore, one of the main challenges is whether they can truly replicate the judgments of human annotators, especially in areas that require understanding context in text analysis. Human moderators can be experienced at identifying forms of manipulation, sarcasm, and irony, and understanding the social and cultural contexts in which misinformation exists. In contrast, language models may struggle with these tasks because, while they can process vast amounts of data, they lack the understanding of context and intent that human moderators have [20,21].

Additionally, these models can be biased by the data they are trained on, leading to potentially inaccurate judgments, similar to the biases that can affect human moderators. Concerns also arise regarding their ability to distinguish between fact and opinion, especially when identifying intent or evaluating source credibility. However, this challenge is not unique to LLMs. Human moderators also struggle with these complexities, as biases and subjective interpretations can influence their judgments. Therefore, understanding the limitations and potential biases of both humans and LLMs is important in developing effective misinformation detection strategies [22,23].

Moreover, detecting and analyzing misinformation is not only about identifying false statements. It also involves understanding the intent behind the content and the linguistic features that may influence how information is perceived and shared [24]. Cognitive analysis of social media users’ posts, which examines the psychological and social factors affecting user behavior, is essential in this context. Traditional computational approaches have struggled to evaluate these aspects due to often focusing on surface-level features without exploring the underlying motivations or implications. The integration of advanced language models presents an opportunity to enhance this understanding, but it also poses many challenges that need to be addressed.

While previous studies have examined the capabilities of LLMs in various aspects of misinformation detection, there is still a gap in understanding how these models perform in cognitive reasoning tasks compared to human annotators. Specifically, there is limited research on the ability of LLMs to interpret user intent, social dynamics, and other cognitive aspects inherent in misinformation. Our study aims to fill this gap by comparing the performance of LLMs and human annotators in these complex tasks, using different prompting strategies to evaluate their effectiveness.

### Research Objectives and Questions

The primary objective of this study is to investigate the effectiveness of LLMs in detecting and interpreting misinformation and how their outputs compare to human annotators, with a specific focus on tasks requiring cognitive analysis and subjective judgment. By comparing the performance of these models using different prompting strategies, we aim to address the following research questions (RQs):

1. Can LLMs replicate human annotators’ judgments in the context of misinformation detection? (RQ1)
2. What are the comparative strengths and weaknesses of different prompting strategies in improving LLM performance on misinformation detection tasks? (RQ2)
3. To what extent do LLM outputs align with expert panel ratings on items that relate to our cognitive framework? (RQ3)

To address RQ3, we established a cognitive framework consisting of 3 parts: content and cognitive analysis, engagement and perception, and personal perception. We build upon established cognitive and social-psychological models to establish a cognitive framework that conceptually relies on the Reasoned-Action-Approach [25].

Building on these insights, we develop a set of RQs based on a cognitive framework encompassing 3 critical elements: knowledge, intention, and action. This structure clarifies how users encounter, interpret, and ultimately propagate misinformation. Adapted from earlier theoretical models, our framework posits that a user’s initial knowledge, shaped by cognitive biases and prior beliefs, guides their intention (whether to share, ignore, or challenge content), which in turn informs their subsequent action (ie, reposting, commenting, or liking). By integrating additional factors such as stance, in-group or out-group identity, and reflection, this cognitive framework provides a comprehensive perspective on how psychological motivations, social influences, and contextual features interact within online environments.

1. Content and cognitive questions support our goal of analyzing intent and cognitive areas to identify where LLMs underperform compared to human annotators. This approach aligns with content analysis methodologies [26], which are fundamental to identifying the ideological leanings or affiliations of users. For example, understanding whether a user’s post aligns with specific ideologies or discusses controversial topics allows us to analyze areas of polarization and potential misinformation triggers [4,14]. Additionally, questions exploring the purpose of the user posts (such as inform, persuade, and entertain) provide insights into user motivations and how content might be received or shared [27-29].
2. Engagement and perception questions enable evaluation of virality and influence, which are critical for understanding the spread of misinformation. These included items relating to engagement patterns, such as virality potential and interaction types (likes, shares, and comments) [30]. These factors provide context for identifying why certain content has virality and how misinformation propagates [31]. Moreover, we examined how users’ posts may influence audience attitudes and behavior by evaluating user stance, persuasive elements, and hidden messages [32]. We included questions exploring manipulative intent or opinion-changing attempts to investigate ethical concerns and the potential impact of misinformation [27].
3. Personal reaction questions address the subjective nature of misinformation analysis. This enables us to identify the interpretive capabilities of LLMs. Questions examining personal reactions and discrepancies between intent and language can show discrepancies and biases [12]. These insights are important for understanding audience response and identifying risks [33]. For instance, exploring whether content appears trustworthy provides context for evaluating its societal implications.

### Related Work: Traditional Approaches to Misinformation Detection

Detecting misinformation in online systems has been a prevailing challenge. Early methods primarily used feature-based machine learning models relying on linguistic characteristics, sentiment analysis, user metadata, and network patterns of information spread. These supervised models required data labeled by humans and domain knowledge for training. While they could detect indicators of misinformation, they struggled to interpret context, intent, and features such as humor or sarcasm that contribute to misleading content [11,13,34].

A significant limitation of these traditional methods is their lack of generalizability across different contexts. Heavily dependent on training and specific features in classification models, they often become outdated as social media platforms and misinformation rapidly evolve. Moreover, variations in misinformation across languages and different social media platforms presented challenges, as models trained on 1 dataset may fail on others [11]. These drawbacks have led researchers to develop more robust and adaptable solutions capable of handling the dynamic nature of social media and the diverse features of misinformation.

### Human Annotation and Moderation

Despite the scalability of automated systems, human expertise remains essential for complex tasks requiring an understanding of context and intent in both model training and moderation. Human moderation to label social media’s extensive content is expensive and time-consuming [18]. Even experienced annotators can disagree on classifying content when definitions of misinformation are unclear or intent is difficult to determine, raising concerns about the reliability of human-annotated datasets used as ground truth. To mitigate these concerns, methods such as consensus building among annotators, clear guidelines, and training have been implemented to improve the reliability of human-annotated datasets [26,35].

### Evolution of LLMs in Misinformation Detection

The development of LLMs has significantly advanced natural language processing. Early models such as GPT-2 [36] showed that unsupervised pretraining on large text corpora, followed by task-specific fine-tuning, could enhance performance in text classification and summarization. Successors such as GPT-3 and GPT-4 [19,37] further demonstrated the potential of LLMs in misinformation detection. Models such as GPT-3 introduced the concept of few-shot learning and helped demonstrate that even a small number of examples can effectively guide large-scale model predictions [19]. However, research also shows that the ordering of prompts can substantially impact few-shot performance, which shows a particular sensitivity in LLMs [38]. These models are suited for detecting misinformation due to their ability to process extensive amounts of text and identify latent patterns. They perform well even without extensive labeled data, making them adaptable to new forms of misinformation, which is a significant advantage over their predecessors to avoid constant retraining [37].

Several studies have applied LLMs to misinformation detection. Wang [39] and Wang et al [40] developed models to detect machine-generated fake news by capturing the structure and style of manipulations within text. Other research has shown that LLMs can outperform traditional models in detecting rumors, conspiracies, and politically motivated misinformation by recognizing deeper semantic patterns and linguistic features [41]. These advancements are relevant to our study as we explore the capabilities of LLMs in detecting misinformation and understanding the underlying cognitive and linguistic features, comparing their performance to human annotators.

However, LLMs can generate hallucinations, which can be defined as possible but incorrect or misleading information [42]. Therefore, models might inadvertently reinforce the misinformation they aim to detect. Moreover, careful prompt design is required to ensure outputs align with initial requirements. Recent advances in prompting strategies, such as chain-of-thought prompting [43], have shown promise in enhancing reasoning capabilities, but their effectiveness compared to human annotators remains to be determined.

### Comparative Studies of Human Annotators and Advanced Models

Research increasingly compares LLMs’ performance with human annotators, particularly in tasks requiring subjective judgment and cognitive analysis. A key advantage of these models is their ability to process large datasets rapidly, analyzing thousands of social media users’ posts in real time to identify misinformation and its linguistic features. However, human annotators are still better at interpreting context and other linguistic features. For example, Thomas et al [20] found that while advanced models can assist human moderators in content moderation tasks, they primarily assist rather than replace human decisions. It remains uncertain whether models can fully replicate human-level analysis in tasks requiring subjective judgment, such as exploring intent or interpreting sarcasm [22].

Some studies show that advanced models have the potential to independently perform tasks traditionally requiring human expertise. Wang et al [40] used BERT and fine-tuned RoBERTa to detect AI-generated news by GPT. The model performed comparably to humans in straightforward factual evaluations but fell short in analyzing advanced linguistic tasks, including interpreting rumors. Similarly, Thomas et al [20] conducted a study demonstrating that LLM-assisted human raters had increased accuracy in content moderation. However, the study only focused on assisting humans rather than directly comparing LLM outputs to human annotations. We interpret this as LLMs may show promise for automating large-scale misinformation detection, but their performance on tasks requiring deeper contextual understanding and subjective judgment remains unclear.

Moreover, recent work has also pointed out the application of LLMs for explainability across a range of health-related domains. One study evaluated ChatGPT’s (OpenAI) reliability in self-diagnosis contexts using medical licensing-style context prompts assessed by both experts and nonexperts. The results showed that while some responses were robust to input variation, only a minority of outputs were consistently rated as factually correct, raising concerns about ambiguity and misinformation in unmoderated use cases [44]. Other research has compared LLM performance in detecting health-related rumors, showing high surface-level accuracy in binary classification tasks, but showing certain limitations in the depth and consistency of explanations, particularly across languages and cultural contexts [45].

Finally, these studies highlight both the potential and limitations of LLMs for misinformation detection, emphasizing the importance of retrieval grounding, careful prompt design, and transparent justification generation in high-stakes applications such as public health.

## Methods

### Ethical Considerations

Our study contributes to the ongoing discussion on the application of computational methods in content moderation. The potential of LLMs to scale misinformation detection is significant, but their deployment raises several ethical concerns. First, there is the risk that these models might reinforce existing biases, given their sensitivity to biases in training data, similar to human biases. Second, the lack of transparency in LLM decision-making processes can lead to challenges in accountability. This raises a fundamental question: how do we ensure that these systems make fair and unbiased judgments, especially when dealing with controversial content? [46].

This study received approval from the University of Bristol Faculty Ethics Committee (ref 18316).

### Dataset

We used a subset of users’ posts from the MuMiN dataset [10], which is a large-scale, multilingual, and multimodal dataset for misinformation detection. We selected a balanced set of users’ posts containing both misinformation (88%) and factual (12%) content to ensure a diverse representation of topics.

### Recruitment and Selection of Human Annotators

We recruited 25 participants (both undergraduate and postgraduate) from the University of Bristol (both undergraduate and postgraduate) through promoting our research through emails. The selection of annotators was based on responses to a screening questionnaire designed to capture participants’ background and suitability for the task. The questionnaire collected information on factors including participants’ experience with X (ie, the frequency of usage and time spent on this social media platform), age, educational background, political orientation, and familiarity with social media platforms. We selected participants who met specific criteria out of an initial 560 respondents, including active users with a minimum of 1 year of experience using X, a range of ages, educational backgrounds, and political affiliations to ensure diversity, varied political orientations to capture different perspectives, and demonstrated familiarity with social media content.

Each annotator evaluated 60 users’ posts and provided three pieces of information for each post: (1) classification decisions on misinformation and cognitive features (5-point Likert-type questionnaire); (2) a self-reported confidence score indicating how certain they felt about their classification, using a 5-point scale (1=very uncertain to 5=very certain); and (3) open-ended justifications for their answers. The annotation questions are provided in Table S1 in Multimedia Appendix 1, and the annotation form instructions and example questions are provided in Section S1 in Multimedia Appendix 1.

Partial responses were excluded from the analysis to maintain data integrity.

### LLM Specifications

#### Overview

We designed an ablation study in which we compared various versions of OpenAI’s GPT series of models, specifically GPT-3.5, GPT-4, GPT-4o, and GPT-4o mini (Table 1) to human annotators. We also explored various prompting strategies and how these affect the performance of the models in our study. We measured performance using precision, recall, specificity, and accuracy. These models were set up to mimic the annotation form provided to human annotators.

**Table 1:** Summary of GPT model versions—overview of GPT model versions, ranging from GPT-3.5 to GPT-4 versions. This table indicates the model release dates and versions.

Model	Version
GPT-3.5	gpt-3.5-turbo-1106
GPT-4 Turbo	gpt-4-turbo-2024-04-09
GPT-4o	gpt-4o-2024-08-06
GPT-4o Min	gpt-4o-mini-2024-07-18

#### Prompting Strategies for LLMs

Recent studies show that refined prompt engineering can substantially improve model outputs [41]. We used 0-shot, few-shot, and chain-of-thought prompting techniques (described in Table 2; example prompts are provided in Table S2 in Multimedia Appendix 1) to generate responses with little or no prior information, checking how well LLMs could apply their existing knowledge to generate responses. However, other prompt strategies, such as batch prompting or a prompt pattern catalog, are not used in this paper [41,47,48].

**Table 2:** Summary of prompting techniques—overview of 3 primary prompting techniques (0-shot, few-shot, and chain-of-thought). This table highlights their descriptions and how each method guides an LLM’sa response generation.

Prompting technique	Description
Zero-shot prompting	Give the LLM a single instruction without any examples related to the task. This method tests the model’s ability to use what it already knows to respond to new prompts, showing how well it can apply its existing knowledge to generate responses [18].
Few-shot prompting	Provide the LLM with examples related to the task before giving it the test prompt. This approach is designed to help shape the model’s response by using examples to guide its reasoning. It depends on the model’s capacity to adapt its answers based on the specifics provided.
Chain-of-thought prompting	This method involves creating instructions that help the LLM to think step-by-step, similar to how annotators would logically tackle these questions. The method helps the LLM to explain each part of its thinking process, improving the accuracy and relevance of the answers it provides.

^a^LLM: large language model.

#### Temperature Control for LLMs

To keep the results consistent and predictable, we set the temperature parameter to 0 for all tests. This helped reduce randomness in the responses that were produced, making the outputs more reliable.

### Ground Truth for Misinformation Detection

In our study, we use binary MuMiN’s misinformation label as the ground truth for the first stage of this study. These MuMiN labels were established by results from multiple fact-checkers, which were standardized by a fine-tuned RoBERTa model, and therefore provide a verified reference for whether a post is considered misinformation. We compared LLM and human annotator performance against this pre-existing ground truth misinformation label.

### Ground Truth for Cognitive Analysis

#### Overview

Human annotator ratings were the reference standard against which we compared LLM performance for cognitive analysis. Given the fact that external fact-checking mechanisms do not exist for cognitive questions, and that there is inherent subjectivity and variability among human raters in such tasks, four different approaches or panel configurations were used to approximate ground truth: (1) precision-based selection, (2) conformity-based selection, (3) clustering technique, and (4) hybrid approach (described further below). Each of these was compared to the various LLM specifications to indicate model performance.

#### Precision-Based Selection

We selected annotators with high precision in detecting misinformation, benchmarked against the MuMiN misinformation label. Precision was measured as the proportion of correctly categorized users’ posts out of the total reviewed.

Therefore, we added a “misinformation label” column to our dataset. This label was determined based on annotators’ scores: if an annotator scored a user’s post 4 or higher on a 5-point Likert scale, the user’s post was labeled as misinformation (misinformation label=1); otherwise, it was not labeled as misinformation (misinformation label=0). We then calculated the precision for each annotator, which was the proportion of users’ posts they correctly identified as misinformation out of all the users’ posts they reviewed. Annotators with a precision score above 65% of the posts that they reviewed were considered reliable in identifying misinformation in most cases.

#### Conformity-Based Selection

For this method, we assume that frequent alignment with the majority reflects accuracy. We calculated the majority response (mode) for each user’s post based on all annotator responses. Therefore, we have chosen annotators who consistently aligned with the majority response for each X post (agreement >65%).

#### Clustering Technique

K-means clustering was used to segment annotators’ responses, self-reported confidence scores, and textual justifications (vectorized using GPT 4o). We used the K-means clustering algorithm, determining the optimal number of clusters using the elbow method. Each cluster’s accuracy was evaluated by comparing the proportion of correct versus incorrect misinformation identifications within the cluster. Clusters with a majority of correct identifications were labeled as high-accuracy. The elbow method was used to determine the optimal number of clusters for grouping our annotators based on their responses and characteristics. This study indicated that 3 clusters (k=3) were the most suitable choice for balancing within-cluster similarity and between-cluster differences. The accuracy of these clusters varied, reflecting the diversity in annotator reliability and perspective (Table 3).

**Table 3:** Cluster performance—this table presents the accuracy of 3 annotator clusters in identifying misinformation.

Cluster	Results
0	Moderate accuracy at 54.07%, indicating a mixed level of performance (no better than chance)
1	The highest accuracy at 71.49%, suggesting a strong ability to identify misinformation consistently
2	The lowest accuracy at 39.12%, which might reflect either a lack of understanding of the misinformation criteria or a differing interpretation compared to other annotators

#### Hybrid Approach

This analysis used the insights from the 3 previous methods to explore whether a combined approach would enhance the accuracy of the misinformation labels. By integrating the precision-based, conformity-based, and clustering results, we aimed to leverage the strengths of each to compensate for its individual limitations. Therefore, the intersection of annotators identified across the 3 methods was used to form the final panel.

The evaluation of accuracy across different panels of annotators was important in determining the effectiveness of each method for identifying misinformation. In our comparative analysis of annotator panels, we have summarized findings that show differentiated performance in Table 4. These findings indicate a combined-methods approach as the most effective strategy, and we will be using this as the main panel of (n=18) human annotators, against which we will compare LLM performance.

**Table 4:** Performance evaluation of different selection methods—this table displays performance metrics for 4 different selection methods of the expert panel.

Study	Description	Precision	Recall	*F*_1_-score	Note
1	Precision-based selection	0.714	0.892	0.793	Panel 1 achieved high recall but lower precision, indicating strong detection capabilities accompanied by a higher occurrence of false positives.
2	Conformity-based selection	0.710	0.872	0.783	Panel 2 displayed reduced performance across all metrics, suggesting that conformity to majority opinions does not necessarily improve detection accuracy.
3	Clustering-based method	0.727	0.903	0.806	Panel 3 improved both precision and recall, showing the effectiveness of a profiling-based approach that leverages individual strengths.
4	Hybrid approach	0.725	0.918	0.810	Panel 4 recorded the highest recall and *F*_1_-scores, effectively minimizing false negatives and demonstrating superior overall performance in misinformation detection.

#### Misinformation Detection Performance

We evaluated each LLM and human annotators on metrics including precision, recall, accuracy, and *F*_1_-score in identifying misinformation in our MuMiN’s ground truth. The results from this evaluation helped us select a final panel of human annotators, which we then used to establish the “ground truth” reference for the cognitive analyses study as described in the next section.

### Comparative Analysis of LLMs

A comprehensive comparison of various LLM models to evaluate their performance across a range of prompting mechanisms was conducted. Using the 0-shot prompt approach as the baseline, the McNemar test was applied to statistically assess the performance of different prompt strategies within each model. This investigation focused specifically on the models’ capabilities to handle complex cognitive questions. Performance metrics were used, such as the *F*_1_-score, precision, and recall, to provide a detailed assessment of each model’s ability to interpret the same questions as the panel annotators’ baseline. Hence, the following performance metrics were used: accuracy, precision, recall, and *F*_1_-score to evaluate both human annotators and LLMs.

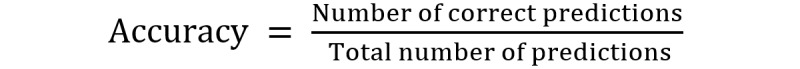

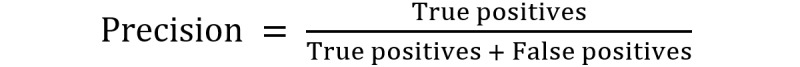

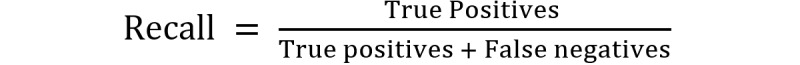

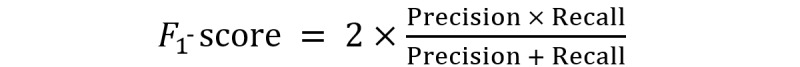

### Categories for Cognitive Analysis of Misinformation

#### Overview

We explored the capabilities of LLMs compared to human annotators using specific cognitive categories (Table 5). These categories were designed to capture various aspects of human cognitive and social dynamics that help understand and identify misinformation and intent.

We aimed to find how LLMs performed compared to human annotators in each cognitive category. This categorization allowed us to analyze the technical aspects, such as linguistic and cognitive features, which contribute to the spread and understanding of misinformation, for example, how individuals interpret misinformation, and how we can evaluate intent and social dynamics of how echo chambers could be formed [49].

**Table 5:** Cognitive categories for misinformation analysis—this table presents the cognitive framework used for the analysis of misinformation in this thesis. These categories enable a detailed comprehension of misinformation ranging from individual cognitive processes to social interactions.

Category	Description
Content analysis	Analyzing the factual accuracy, logical consistency, and credibility of the content itself
Perception and intent	Assessing the underlying intentions of the content creator and how the message is likely to be perceived by other users. This involves interpreting sarcasm, irony, and implicit meanings
User engagement and interaction	Analyzing how users interact with the content, including likes, shares, comments, and the potential for virality. This reflects the content’s influence on different users
Personal reaction	Evaluating the emotional responses that the content may provide in individuals, such as fear, anger, empathy, or trust
Behavioral analysis	Investigating patterns in user behaviors that might indicate bot activity or organized activity
Social and group dynamics	Understanding how the content affects or reflects group behaviors, social norms, sentiments, and echo chambers or polarization

#### LLM Ratings

We conducted a comparative analysis of several LLMs on a multilabel classification task involving cognitive questions related to posts from the MuMiN dataset. The models were tasked with generating responses to these cognitive questions, and their responses were evaluated based on multilabel classification criteria. The models analyzed were GPT-4 Turbo, GPT-4o, GPT-4o mini, and GPT-3.5. We provided the same questions that annotators answered, and to enhance fairness. We configured all models with a temperature setting of 0. This setting minimizes randomness in the model’s answer generation processes, allowing for consistent responses across multiple runs.

#### Confidence Scores

This section highlights the relationship between the confidence scores provided by LLMs and their accuracy. We analyzed the self-reported confidence metrics of the models. Confidence scores represent the probability assigned by the LLM to its chosen prediction, which indicates the model’s confidence about the correctness of its response. While these scores are not direct measures of reliability, they provide insights into the model’s self-assessment capabilities.

Our analysis examines how well confidence scores aligned with prediction accuracy across different cognitive categories. Furthermore, we calculated the Pearson correlation coefficient between the confidence levels and the correctness of the predictions for the following categories: content analysis, user engagement and interaction, personal reaction and perception, and intent recognition.

### Case Study Selection and Analysis

We conducted a focused case study analysis on selected posts in order to show differences between LLMs and human annotators. We identified posts from the annotated MuMiN subset that have shown particularly interesting features such as sarcasm, subtle health misinformation, or ambiguous intent. We then tried to examine how both LLMs and human annotators labeled these posts regarding their misinformation label and the cognitive categories of perception and intent, user engagement, and personal reaction.

## Results

### Detecting Misinformation: Comparing LLM and Human Annotator Performance

#### Overview

Table 6 shows the performance metrics of different GPT models against the ground truth misinformation labels that we extracted from the MuMiN dataset. Among the models we tested, GPT-4 Turbo achieved the highest accuracy of detecting misinformation: 67.2%, with a precision of 75%, a recall of 81.8%, and an *F*_1_-score of 78.2%. In comparison, GPT-3.5 achieved an accuracy of 63.93%, precision of 84.3%, recall of 61.3%, and an *F*_1_-score of 71%.

Comparatively, the panel of annotators achieved an accuracy of 70.1%, precision of 72.5%, recall of 91.8%, and an *F*_1_-score of 81%. These findings have important implications for future content moderation and cognitive understanding of LLMs. While human annotators showed slightly higher accuracy and recall, GPT-4 Turbo’s comparable performance in terms of precision and *F*_1_-score suggests it can be used as a scalable alternative for moderation, especially in areas requiring high precision, such as misinformation detection. This also points to the potential for hybrid systems, where GPT-4 handles large-scale initial sets of users’ posts, and human moderators focus on supervision and validation. Such an approach could optimize resource efficiency while maintaining high-quality outputs. This result underscores the evolving role of human moderators in complementing AI systems and calls for further exploration into collaborative frameworks that maximize the strengths of both.

**Table 6:** Performance metrics of different GPT models against MuMiN’s ground truth—this table shows the accuracy, precision, recall, and F1-scores for 4 LLMa-based models and a panel of human annotators (expert panel) when compared against MuMiN’s ground truth.

Model	Accuracy (%)	Precision (%)	Recall (%)	*F*_1_-score (%)
GPT-3.5	63.93	84.38	61.36	71.05
GPT-4 Turbo	67.21	75	81.82	78.26
GPT-4o Mini	60.66	81.25	59.09	68.42
GPT-4o	62.3	76.92	68.18	72.29
Panel of annotators	70.1	72.5	91.8	81

^a^LLM: large language model.

#### Analysis of Prompting Strategies

We evaluated the effect of different prompting strategies (0-shot, few-shot, and chain-of-thought) on the performance of the GPT models. Table 7 presents the performance metrics for GPT-4 Turbo under these prompting conditions.

The chain-of-thought prompting strategy yielded the best performance, with an accuracy of 72.1%, precision of 78%, recall of 85%, and an *F*_1_-score of 81%. Such findings suggest that providing the model with a structured reasoning path enhances its ability to interpret complex cognitive questions.

We applied the McNemar test to explore the statistical significance of the differences between prompting strategies. The test results indicated that the performance improvement of chain-of-thought prompting over 0-shot prompting was statistically significant (*P*<.05).

**Table 7:** Performance of GPT-4 Turbo for different prompting strategies—this table presents GPT-4 Turbo’s performance under 3 prompting strategies (0-shot, few-shot, and chain-of-thought) across accuracy, precision, recall, and F1-score.

Prompting strategy	Accuracy (%)	Precision (%)	Recall (%)	*F*_1_-score (%)
Zero-shot	65.57	73	79	75.9
Few-shot	68.85	76.5	82	79.13
Chain-of-thought	72.13	78	85	81.33

#### Confidence Scores of LLMs and Accuracy

Our findings illustrate a positive correlation between confidence scores and prediction accuracy in the areas of content analysis (ρ=0.72, *r*<0.01) and user engagement and interaction (ρ=0.65, *r*<0.05). These results suggest that in straightforward tasks with simpler linguistic patterns, higher confidence scores correspond to greater accuracy. For example, they were both confident and correct in cases where the models analyzed clear misinformation statements.

In contrast, the correlation was weaker in the area of personal reaction and perception (ρ=0.38, *r*=0.12) and intent recognition (ρ=0.33, *r*=0.15). These categories involve subjective and more cognitive interpretation, such as understanding sarcasm or underlying intent, where the models expressed overconfidence in incorrect predictions. This difference indicates that while confidence scores can be used for reliability in certain contexts, they fail to benefit in more complex analyses.

### Cognitive Categories

#### Overview

We further analyzed the models’ performance across different cognitive science categories. Table 8 compares the best GPT-4 Turbo and worst-performing LLM GPT-3.5 in terms of accuracy, precision, recall, and *F*_1_-score for each category. Overall, GPT-4 Turbo outperformed GPT-3.5 across all categories, particularly in user engagement and interaction and social and group dynamics. However, performance in categories including perception and intent and personal reaction remained low for both models, indicating challenges in capturing human interpretations in these areas.

**Table 8:** Comparison of GPT-4 Turbo versus GPT-3.5 for cognitive categories—this compares GPT-4 Turbo and GPT-3.5 across 6 cognitive categories, showing higher accuracy, precision, recall, and F1-scores for GPT-4 Turbo in every category. Overall, GPT-4 Turbo consistently outperforms GPT-3.5 based on these metrics.

Category and model		Accuracy (%)	Precision (%)	Recall (%)	*F*_1_-score (%)
Content analysis					
	GPT-4 Turbo	41.4	43.3	41.4	42
	GPT-3.5	20.9	46	20.9	22.6
Perception and intent					
	GPT-4 Turbo	23.8	47.7	23.8	24.7
	GPT-3.5	11.1	36.2	11.1	10.5
User engagement and interaction					
	GPT-4 Turbo	63.4	69.6	63.4	66
	GPT-3.5	58.7	65.4	58.7	61.8
Personal reaction					
	GPT-4 Turbo	24	42	24	30
	GPT-3.5	19.7	41.6	19.7	26.6
Behavioral analysis					
	GPT-4 Turbo	31.1	26.3	31.1	28.5
	GPT-3.5	21.9	25.6	21.9	23.6
Social and group dynamics					
	GPT-4 Turbo	45.9	67.4	45.9	54.9
	GPT-3.5	13.9	36.4	13.9	20

#### Comparison With Human Annotators

We compared each LLM’s outputs to the labels from our final panel of 18 annotators for each cognitive category.

Our results show that human annotators consistently outperformed the models in tasks requiring deeper contextual understanding and subjective judgment. For instance, in perception and intent, the panel consensus achieved an accuracy of approximately 60% when compared within themselves (eg, comparing each annotator’s label to the vote within the final panel), whereas GPT-4 Turbo’s accuracy measured against that same panel was only 23.8%. This gap highlights the complexity of interpreting context, sarcasm, or hidden intent.

Importantly, “accuracy” for human annotators in these cognitive labels was based on how often each individual aligned with the final panel. This ensures consistency in evaluating both LLM and human performance, though we acknowledge that the final panel’s answers themselves may still contain subjectivity.

## Discussion

### Principal Results

Based on the statistical significance established in our analysis, we explore the implications of these findings in the following discussion. As intent remains difficult to establish in online content, both human and LLM evaluations must be treated as interpretive rather than conclusive. Detecting misinformation on X is very challenging due to complex content, social interactions, and huge amounts of data. We conducted a comparative study on how well LLMs and human annotators detect misinformation, focusing on how they understand behavior and context. While our cognitive framework aims to explore how LLMs and humans interpret features such as intent and perception, we acknowledge the limits of establishing these features only from textual content. Our findings should be interpreted as an investigation and comparison with a panel of experts. These limitations reflect broader challenges also faced by human moderators and platforms when interpreting user intent under content moderation policies.

A hybrid approach, where computational models work alongside human moderators, may offer the most effective solution. Such a system could improve the scalability of content moderation while relying on human expertise for more complex cases that require ethical consideration [11]. Our results reveal important insights into what LLMs can and cannot do, suggesting ways to combine machine learning models with human judgment in content moderation systems.

### Comparing Performance Across Different LLMs

Our analysis revealed that the chosen model of LLM significantly impacted performance in misinformation detection tasks. GPT-4 Turbo outperformed chosen models across all metrics, demonstrating higher accuracy, precision, recall, and *F*_1_-score. For instance, GPT-4 Turbo achieved an accuracy of 67.21%, while GPT-4o and GPT-4o mini achieved 62.30% and 60.66%, respectively. This trend indicates that larger models have enhanced capabilities in pattern recognition and contextual understanding.

Moreover, recent benchmark studies have raised important questions about the reasoning capabilities of LLMs. Shojaee et al [50] observed a decline in model performance on structured reasoning tasks at higher levels of complexity, which was interpreted as a sign of cognitive limitation. However, further research by Lawsen [51] and Varela et al [52] suggested that such failures may instead reflect constraints in experimental design, such as unsolvable task configurations or token-length limitations, rather than fundamental reasoning deficits. These findings echo patterns in our own study, where LLMs performed well on certain structured misinformation tasks but diverged from panel annotators on more interpretive items, such as user intent and sarcasm. Importantly, these areas are challenging not only for models but also for human raters, as judgments about meaning, motive, or tone can be inherently subjective. As such, these disagreements may not reflect clear errors but rather underline the difficulty of establishing a consistent ground truth for cognitive analysis. This highlights the need for further research into how both humans and models interpret subtle linguistic cues, and how such interpretations can be evaluated more reliably.

### Strengths and Limitations of LLMs

#### Performance of Chain-of-Thought Prompting

Chain-of-thought prompting generally performed better than other prompting methods, including 0-shot and few-shot across various cognitive areas, but its success varied based on task complexity.

In areas that rely on clear pattern recognition, such as analyzing user engagement and interactions, chain-of-thought prompting has significantly improved both accuracy and recall. This aligns with researchers who suggest treating LLMs as “program executors” where step-by-step reasoning helps reduce errors [53]. By thinking through each step, LLMs were better at identifying strategies that kept users engaged, leading to more precise results. However, chain-of-thought can also lead to “greedy reasoning,” where the model is based on incomplete logic [54].

Yet, in more complicated areas, including understanding perceptions and intentions, the advantages of chain-of-thought prompting were not as strong. Although there was still some improvement, compared to other prompting strategies, the LLMs did not perform as well as they did in the simpler cognitive categories. This indicates that while breaking down the reasoning process helped the LLM organize its tasks to do and improve its reasoning, it does not completely overcome the LLM’s challenges in understanding deeper behavioral questions.

Future studies might look into more advanced prompting methods or combine chain-of-thought with other contextual information (such as prior X posts or followers of each user) to further improve performance in these complex areas.

To conclude, our results indicate that when using chain-of-thought prompting strategies, GPT-4 Turbo nearly matches human performance in several tasks, especially those involving logical reasoning. For example, the model was good at detecting clearly false statements and identifying patterns of how misinformation spreads, using reasoning similar to that of human annotators. However, the models have limitations, especially in areas that require a deeper understanding of behavior and context.

#### Human Annotators’ Strengths: Understanding and Judgment

Human annotators consistently outperformed the models in tasks requiring deep contextual understanding and subjective reasoning. They were better at interpreting aspects of user intent and social dynamics, especially when misinformation was contextual or was written to sound truthful. This supports previous research that emphasizes the importance of human insight in interpreting context, behavior, and intent in social media content [6,18,20].

For example, when users discussed misinformation about diet strategies, human annotators were more skilled at determining the intent behind the message, while models often misread the content as factual or harmless. This highlights the limitations of language models in tasks needing more than basic analysis tasks, where social, emotional, and cultural knowledge are crucial for accurate content classification.

GPT-4 Turbo found tasks involving analyzing intent and linguistic features difficult, even with advanced prompting strategies. For instance, GPT-4 Turbo struggled to detect misinformation in a user’s post that used satire to critique health misinformation, misclassifying it as genuine content. This aligns with previous research showing that while language models handle surface-level analysis well, they often struggle with tasks needing deeper thinking or social understanding. For instance, the models were less effective at identifying misinformation when the intent behind the message was unclear or when context significantly changed the meaning.

#### Comparing Performance Across Cognitive Categories

Our analysis, Table 8, of performance across specific cognitive categories revealed that models such as GPT-4 Turbo performed similarly to human annotators in straightforward categories such as user engagement and interaction and social and group dynamics, where finding obvious patterns was sufficient. However, GPT-4 Turbo’s performance dropped significantly in categories requiring interpretation of perception and intent and personal reaction, where human emotional judgment and cultural awareness were important.

For instance, in the user engagement and interaction category, the models effectively identified users’ posts designed to provoke engagement through provocative or polarizing content, as shown by their relatively high recall scores. However, GPT-4 Turbo struggled in the perception and intent category, which required understanding the underlying intentions of the user who authored the post. Human annotators were better at recognizing when a user’s post was intended to mislead, even if the content was not obviously false.

#### Limitations of LLMs in Contextual Interpretation

We found that LLMs often struggle when it comes to accurately categorizing content related to perception and intent or personal reactions (Table 8).

These areas require a deep understanding of complex human emotions, hidden intentions, and the contexts that are not always clearly stated in the text. For example, telling the difference between someone genuinely expressing concern and making a sarcastic comment involves picking up on tone and underlying motives, which goes beyond just processing the words.

Research has consistently shown that these models have difficulty with tasks that require a theory of mind or cognitive reasoning [55]. While they are good at recognizing and generating text based on patterns they have learned, they do not possess the natural cognitive abilities that humans use to accurately infer intentions and emotional states.

Additionally, LLMs often miss cultural and contextual subtleties, which makes their performance even worse in these categories. For instance, sarcasm that depends on context or idioms specific to a culture can easily be misunderstood, leading to less accurate and reliable classifications. Recent studies support this, highlighting that models frequently overlook the deeper meanings and practical aspects of human communication. Understanding these challenges is crucial for improving how LLMs analyze cognitive tasks. It shows the importance of incorporating more advanced learning methods, such as using multiple types of data and better context-aware techniques, to create a richer understanding of complex human reasoning.

Finally, we conducted a manual review of LLM outputs as part of our evaluation to analyze both the Likert-scale classifications and the accompanying open-ended justifications. This hallucination audit aimed to identify cases where the model’s explanations introduced information not present in the original user post, a phenomenon known as hallucination. While hallucinations were more evident in the open-ended responses, where the LLM occasionally inferred motivations or context beyond the given text, they may also influence binary or scaled answers, particularly when these judgments rely on false information. These examples suggest that hallucinations may subtly shape LLM classifications. This limitation offers a possible explanation for divergences between LLM and human annotator decisions and underscores the need for caution when interpreting LLM outputs in high-stakes settings such as misinformation detection.

### Case Studies

#### Overview

We present 3 detailed case studies involving users’ posts where all LLMs underperformed in order to show the limitations of LLMs compared to human annotators in detecting misinformation (Table S3 in Multimedia Appendix 1). Our analysis of human annotators’ and LLMs is based on the provided justification by them.

#### Example 1: Political Sarcasm

Human annotators’ justifications indicated that they interpreted the selected user post as discussing misinformation, primarily due to its reference to unverified and potentially false allegations concerning a political figure. Several annotators noted a sarcastic tone and perceived the content as suggestive of corruption, which they considered potentially misleading in the absence of credible evidence. This interpretation reflects the broader patterns observed in the Results section, where human annotators showed some sensitivity to rhetorical features such as sarcasm and indirect implication.

In contrast, the LLM did not flag the post as misinformation (example 1). Instead, it appeared to interpret the content as a general political opinion or criticism, without identifying the underlying tone or indirect implications. This may illustrate some of the model’s limitations in capturing specific language features, including sarcasm or rhetorical framing. While the model’s output followed a coherent line of reasoning based on the surface content, it may have lacked the contextual depth to infer possible intent.

This example suggests that LLMs may face challenges in identifying indirect or implied misinformation, particularly in politically charged discourse where tone, context, and social knowledge are key to interpretation. It also points to the continuing value of human moderation in complex analytical tasks, especially where judgments about user intent or potential societal impact are involved.

#### Example 2: Health Misrepresentation

Human annotators tended to classify this post as discussing misinformation, primarily due to its promotion of unverified and potentially unsafe alternative treatments for serious medical conditions. Several annotators noted that suggesting raw vegetable juice as a cure lacked scientific grounding and could, in their view, discourage individuals from seeking conventional medical care. Their responses reflected concern about the potential implications of such claims in public health contexts.

In comparison, the LLM did not identify the post as misinformation. It appeared to interpret the content as a personal narrative or opinion without further analyzing further public health implications of the treatment being described. This may suggest a limitation in the model’s ability to evaluate persuasive intent or emotionally framed content.

As shown in example 2, this case illustrates some of the challenges LLMs may face when processing health-related narratives that blur the line between personal testimony and misinformation. The model’s output, while internally coherent, did not reflect the same level of caution observed in human annotator responses. This difference underscores the importance of continued human involvement in moderation processes, particularly in domains where the potential for harm is closely tied to misinterpretation of health advice.

#### Example 3: Health

In example 3, human annotators generally classified this post as discussing misinformation or potentially false claims related to pregnancy. Some annotators expressed concern that the information shared in the post could cause confusion or cause concern among users, particularly those seeking guidance on maternal health. Their interpretations reflected a cautious approach to unverified claims in sensitive health contexts.

The LLM, however, did not flag the post as misinformation. It appeared to interpret the content as an expression of personal surprise or curiosity, without further evaluating the credibility of the underlying claim. This may indicate a limitation in the model’s ability to assess the implications of health-related statements that are framed informally or anecdotally.

Therefore, LLMs could struggle to identify potentially misleading health content when it is hidden through indirect or emotionally neutral language, based on this example. While human annotators tended to consider the broader context and possible impact on readers, the model focused more narrowly on surface features of the text. Our results imply that additional safeguards or contextual prompts may be needed when using LLMs to evaluate health-related social media content.

### Relationship Between Accuracy and Confidence of the LLMs

We also looked into how confident the models were about their predictions and whether that confidence matched their actual accuracy. By checking the confidence scores the LLMs provided, we wanted to see if higher confidence meant more accurate results, which would help us understand how much we can trust the models’ self-assessments.

This indicates there is a positive link between confidence and accuracy in most areas, especially in content analysis and user engagement and interaction. In these fields, the models were more likely to be correct when they were more confident in their answers.

Nevertheless, this relationship was not as strong in areas such as personal reaction and perception, and intent. In these more complex categories, the models sometimes felt very confident but were still wrong. This suggests that while confidence scores can be a good sign of reliability in some situations, they are not always consistent across all types of cognitive analysis.

These findings highlight the need to develop better ways to measure confidence that take into account the complexity and subjective nature of certain behaviors. Improving these confidence metrics could make LLMs more trustworthy overall.

### Implications for Misinformation Detection Systems

These findings have important implications for developing and using automated misinformation detection systems. However, misinformation varies in severity from harmful health claims (ie, drinking bleach to cure COVID-19) to political satire. Our findings suggest LLMs perform better in detecting clear factual inaccuracies, especially when tied to immediate public health risk. Conversely, failures in interpreting sarcasm in political discourse may represent lower immediate harm. These should inform platforms’ moderation pipelines, which can deploy LLMs as a first filter for high-risk content, while reserving complex social interpretations for human moderators.

Given limitations as discussed, we suggest that hybrid systems, combining the scalability of language models with the understanding of human annotators, may offer a strong solution for misinformation detection on online platforms.

The models could serve as the first line of defense, flagging potential misinformation based on language patterns, while human reviewers focus on more challenging cases requiring deep contextual understanding and ethical judgment. This approach would improve the accuracy of misinformation detection and ensure that it remains flexible to the diverse and evolving nature of online content. Additionally, future work should consist of fine-tuning LLMs using an extensive dataset that includes labels of cognitive linguistic features. This task could help LLMs improve their performance and focus on these details.

Moreover, prompting strategies such as chain-of-thought have shown promise in narrowing the gap between model and human performance, suggesting that refining these techniques could lead to better results. Incorporating such strategies into misinformation detection pipelines could improve the interpretative abilities of language models, helping them more closely mimic human reasoning in complex cases.

### Strengths and Limitations of This Study

One of the key strengths of this research is its multimethod approach to determining ground truth for both misinformation detection and cognitive categories. The MuMiN dataset provides fact-checking labels for misinformation, and a 3-part method (precision-based selection, conformity-based selection, and clustering) offers a way to identify a reliable panel for the more subjective cognitive labels. This process increases confidence in this study’s findings about LLMs’ capabilities compared with human judgment.

However, this study has some limitations. First, depending on human annotators to assign cognitive labels can introduce subjectivity, even with efforts such as building panels of experts. The final panel may not be a perfect source of truth, since humans can still make mistakes and show bias based on their beliefs and background. Second, the MuMiN dataset might not represent every social media environment, which limits how broadly the results can be applied. Third, real-world examples can involve complex issues such as sarcasm or subtle health claims that may require cultural knowledge.

### Conclusions

Our evaluation of LLMs to analyze their analysis of misinformation compared to human annotators’ outputs revealed insights into their abilities to interpret behavior and context on social media users’ posts.

We examined the impact of different prompting strategies (including 0-shot, few-shot, and chain-of-thought prompting) on model performance across various cognitive categories. Our findings indicate that while LLMs such as GPT-4 Turbo show promising capabilities and can approach human performance in tasks involving logical reasoning and pattern recognition, they are still behind in areas requiring a deep understanding of human behavior, context, and cultural understanding. However, chain-of-thought prompting improves model performance by guiding models through reasoning processes, suggesting that such strategies are beneficial. By conducting this comprehensive evaluation, our study significantly contributes to the understanding of LLM capabilities in the area of misinformation detection and cognitive analysis. These insights highlight the potential of LLMs as tools to support human moderators but also show the need for continued human involvement in misinformation detection.

### Future Work

Future work could focus on fine-tuning LLMs with datasets that emphasize cognitive and emotional linguistic features to enhance their understanding of complex human behaviors. Exploring new prompting strategies, such as integrating chain-of-thought with additional contextual features of multimodal data, may further improve model performance. Finally, users’ engagement with social media posts may be influenced by both textual and visual elements, making misinformation detection more complex. Additionally, integrating ethical considerations into automated systems will be crucial for developing responsible and effective misinformation detection tools.

## Appendix

Multimedia Appendix 1 Data on annotations, prompts, and malinformation.

## Abbreviations

AI: artificial intelligence
LLM: large language model
RQ: research question
