# Supplementary material for: Performance of Large Language Models in the Cognitive Analysis of Misinformation: Evaluation Study
Source: JMIR Infodemiology. 2026 May 18;6:e72524. doi: 10.2196/72524 (PMC13227085; doi:10.2196/72524)

**Table S1.** Annotation question.

| Category | Question |
| --- | --- |
| Content Analysis | Q1: Does the spreader’s profile description contain content from specific sources or ideological leanings?  Q7: Does the user's post discuss highly controversial topics?  Q13: This user's post discusses misinformation.  Q14: What do you believe was the primary purpose of the user's post?  Q18: Do you think the user's post was premeditated or spontaneous? |
| Social and Group Dynamics | Q4: Does the user associate themselves with any political or social group that might influence their sharing behaviour based on their profile description?  Q5: Are there indications that the spreader is sharing the information to defend or promote their group’s status?  Q15: Do you think the user's post was intended for a broad audience or a specific group of followers? |
| Cognitive Analysis | Q2: What emotions can you identify in this user's post?  Q17: Assess the tone of the user's post.  Q23: Does the user's post seem to be trying to make you feel certain emotion? |
| User Engagement and Interaction | Q8: Has this user's post gone viral, in your opinion?  Q9: Engagement  Q11: Does the user's post ask for direct engagement in their user's post? |
| Perception and Intent | Q6: Stance  Q10: Persuasiveness  Q19: Is there any hidden message which could be interpreted differently by various groups?  Q20: Could the user's post be perceived as manipulative?  Q22: Do you feel that the user's post was trying to change your mind on something? |
| Personal Reaction | Q16: Is there a discrepancy between the apparent intent of the user's post and the language used?  Q21: What was your immediate reaction to this user's post?  Q24: Is the user's post suggesting that you don't do something or ignore a particular issue?  Q25: Do you think this user's post could start an argument?  Q26: Does this user's post seem trustworthy to you? |

**Section S1.** Example of annotation form questions.

Instructions: You can annotate a set of 10 user's posts using this annotation form.

1. Please take a break before proceeding to another set of 10 user's posts.
2. These user's posts **might contain false information**.
3. Please **do not** reshare them or believe in their content. They are only used for research purposes.
4. Additionally, please avoid checking any information about these user's posts, as this could introduce bias into the study
5. Some questions might not be relevant to the tweet. (i.e. questions about user description when the user description is not available) In this case, please choose "Strongly Disagree" and mark your confidence as (1) low confidence.
6. **Question 15a** was incorrectly set up. Please choose 1 for a broad audience and 5 for a specific group of followers.
7. Open-text questions are **non-mandatory**. Please put N/A if you don't want to answer.
8. Please don't forget to submit your answers; this is the only way we can receive your answers. The "Submit" button is located at the end of this form.
9. This task should take between 1h -1h30 minutes.
10. Please analyse only provided user description and tweet text.
11. Questions which are like 'Tweet ID: <tweet_number>' are an admin questions.

*Please be advised that any data submitted through this form regarding future participants will be securely deleted if they are not selected for participation. Our priority is to maintain confidentiality and integrity in the handling of your data. By submitting this form you also agree to the Terms and Conditions of Google Forms which is used to submit and process this form.*

Annotator_ID: <unique_id>

Tweet_ID: <user_post_id>

User Description: Progressive, engineer, grandpa, birdWatcher, polyglot,world traveler, Episcopalian, married, loves humans, education booster #FBR #Resist #DemVoice1

Tweet Text: If GOP liars really cared about being prolife, they wouldn’t ignore the number one cause of death among US children, gun deaths or our country’s infant mortality rate. Why do republicans hate American women so much? Women deserve better than a party that only cares about control.

1a. Does the spreader’s profile description contain content from specific sources or ideological leanings?
1b. How confident are you about your answer? (Choose 1-5 on Likert scale where 5 is very confident)
1c. Please provide short justification: (2-3 sentences)

2a. What emotions can you identify in this tweet?
2b. How confident are you about your answer? (Choose 1-5 on Likert scale where 5 is very confident)
2c. Please provide short justification: (2-3 sentences)

3a. Are there any mentions of sharing their own experience (Reflective tone of tweet)?
3b. How confident are you about your answer? (Choose 1-5 on Likert scale where 5 is very confident)

4a. Does the user associate themselves with any political or social group that might influence their sharing behavioural based on their profile description?
4b. How confident are you about your answer? (Choose 1-5 on Likert scale where 5 is very confident)

5a. Are there indications that the spreader is sharing the information to defend or promote their group’s status?
5b. How confident are you about your answer? (Choose 1-5 on Likert scale where 5 is very confident)
5c. Please provide short justification: (2-3 sentences)

6a. Stance of the tweet?
6b. How confident are you about your answer? (Choose 1-5 on Likert scale where 5 is very confident)
6c. Please provide short justification: (2-3 sentences)

7a. Does the tweet discuss highly controversial topics?
7b. How confident are you about your answer? (Choose 1-5 on Likert scale where 5 is very confident)

8a. Has this tweet gone viral, in your opinion?
8b. How confident are you about your answer? (Choose 1-5 on Likert scale where 5 is very confident)

9a. Engagement indicators?
9b. How confident are you about your answer? (Choose 1-5 on Likert scale where 5 is very confident)

10a. Persuasiveness of the tweet?
10b. How confident are you about your answer? (Choose 1-5 on Likert scale where 5 is very confident)
10c. Please provide short justification: (2-3 sentences)

11a. Does the tweet ask for a direct engagement?
11b. How confident are you about your answer? (Choose 1-5 on Likert scale where 5 is very confident)

12a. Sentiment of the tweet?
12b. How confident are you about your answer? (Choose 1-5 on Likert scale where 5 is very confident)

13a. This tweet discusses misinformation?
13b. How confident are you about your answer? (Choose 1-5 on Likert scale where 5 is very confident)

14a. What do you believe was the primary purpose of the tweet?
14b. How confident are you about your answer? (Choose 1-5 on Likert scale where 5 is very confident)

15a. Do you think the tweet was intended for a broad audience or a specific group of followers?
15b. How confident are you about your answer? (Choose 1-5 on Likert scale where 5 is very confident)

16a. Is there a discrepancy between the intent of the tweet and the language used (informative tweet using highly emotive language)?
16b. How confident are you about your answer? (Choose 1-5 on Likert scale where 5 is very confident)

17a. Assess the tone of the tweet.
17b. How confident are you about your answer? (Choose 1-5 on Likert scale where 5 is very confident)

18a. Do you think the tweet was premeditated or spontaneous?
18b. How confident are you about your answer? (Choose 1-5 on Likert scale where 5 is very confident)

19a. Is there any hidden message which could be interpreted differently by various groups?
19b. How confident are you about your answer? (Choose 1-5 on Likert scale where 5 is very confident)

20a. Could the tweet be perceived as manipulative?
20b. How confident are you about your answer? (Choose 1-5 on Likert scale where 5 is very confident)

21a. What was your immediate reaction to this tweet?
21b. Do you feel that the tweet was trying to change your mind on something?
21c. Does the tweet seem to be trying to make you feel certain emotion?

22a. Does the tweet suggest that you don’t do something or ignore an issue?
22b. Could this tweet start an argument? (Choose 1-5 on Likert scale where 5 is very confident)
22c. Does this tweet seem trustworthy to you? (Choose 1-5 on Likert scale where 5 is very confident)

**Table S2.** Example of the prompts - These tables (A-C) show three prompts setups for classifying user posts by cognitive framework regarding online misinformation. Each approach provides different instructions, examples, and justifications for how to output a Likert score, confidence level, and reasoning.

1. Zero-shot Prompt

| You are provided with user's post text and user description below. Question: Does this user's post discusses misinformation? Classify the text using a Likert Scale from 1 [Strongly Disagree] to 5 [Strongly Agree]  Please classify your confidence score from 1 to 5 where 5 is very confident.  user's post: While my body is slow to change, it's my face that tells the story. When I saw the first photo back in September, I was 4 months into my carnivore diet. I was dismayed at how old I looked, even though I was feeling so much better already after changing my diet. Fast forward almost two years and I can see that I'm not only feeling younger, I look younger as well. Aging backward is a thing! <hashtag>carnivorediet <hashtag>meatheals <hashtag>antiaging  <hashtag>reverseaging  User Description: Ex vegan, vegetarian. Now carnivore. Natural therapist. Healing my body with a carnivore diet one day at a time. Link tree  Output: Question: [ ]  Confidence: [ ]  Justification: [ ]  ' |
| --- |

1. Few-shot Prompt

| Example 1:user's post "What do you think of the new policy changes? Let us know your thoughts!"  User Description: "Public policy enthusiast, always seeking feedback."  Question:Does this user's post ask for direct engagement?  Classification: 5 [Strongly Agree]  Confidence: 5 [Very Confident]  Justification: The user's post explicitly asks for audience opinions, indicating a clear request for engagement.,Example 2:  user's post: Today, it is an election day.  User Description: Journalist. Historian.  Question:Does this user's post ask for direct engagement?  Classification: 3 [Neither Agree or Disagree]  Confidence: 4 [Confident]  Justification: The user's post mentions an election day but doesn’t ask for audience engagement.,Now analyse the following new user's post:  user's post: While my body is slow to change, it's my face that tells the story. When I saw the first photo back in September, I was 4 months into my carnivore diet. I was dismayed at how old I looked, even though I was feeling so much better already after changing my diet. Fast forward almost two years and I can see that I'm not only feeling younger, I look younger as well. Aging backward is a thing! <hashtag>carnivorediet <hashtag>meatheals <hashtag>antiaging <hashtag>reverseaging  User Description: Ex vegan, vegetarian. Now carnivore. Natural therapist. Healing my body with a carnivore diet one day at a time. Link tree -  Question: Does the new user's post ask for direct engagement? Classify on a scale from 1 to 5.  Provide a confidence score on your classification from 1 to 5.  Output: Question:[ ] Confidence: [ ] Justification: [ ] |
| --- |

1. Chain of Thought

| - Detect any positive, negative, or neutral sentiments.  - Keywords and phrases that indicate emotion, like "happy," "sad," "angry," or "excited," will help the sentiment analysis.  - The overall context of the user's post will help assess the sentiment assessment, considering irony or sarcasm if applicable based on the context  Text +User Description:  Output:  - Question: Determine Likert score based on the overall sentiment of the provided user's post and user description  - Confidence: Asses the confidence based on your clarity of the sentence  - Justification: Explain your decision  User's post: While my body is slow to change, it's my face that tells the story. When I saw the first photo back in September, I was 4 months into my carnivore diet. I was dismayed at how old I looked, even though I was feeling so much better already after changing my diet. Fast forward almost two years and I can see that I'm not only feeling younger, I look younger as well. Aging backward is a thing! <hashtag>carnivorediet <hashtag>meatheals <hashtag>antiaging  <hashtag>reverseaging  User Description: Ex vegan, vegetarian. Now carnivore. Natural therapist. Healing my body with a carnivore diet one day at a time. Link tree  Output: Question: [ ]  Confidence: [ ]  Justification: [ ] |
| --- |

**Table S3.** Examples of user posts discussing political and health misinformation- Table shows chosen examples of users’ posts which discuss political and health misinformation. Each example presents the anonymised user’s bio and their corresponding posted claim.

|  | User's bio | User’s Post | LLM justifications | Participant justification |
| --- | --- | --- | --- | --- |
| 1 | Self- identified as a conservative | Claims a political figure’s income omits bribes or corrupt earnings  (*On this year's tax return, PRESIDENT reported his family’s ADJUSTED GROSS INCOME as $619,976*  *"Adjusted gross income" obviously means adjusted to not include the war grift, the bribes from China, or the big Pharma kickbacks.*) | *“This appears to be political commentary rather than a false claim. It does not clearly present misinformation.”* | The tweet refers to the figures of the president's income from the tax return document. Users make sarcastic statements to imply corruption. |
| 2 | Shares their age and gender, URL link to their store with supplements | Story about using vegetable juice to treat terminal cancer of their daughter.  *(Mom evades authorities (who would medically kidnap baby), takes baby to Mexico for a "food cure." It involves a large amount of raw vegetable juice. 👉http://<URL>.com 🤯 That baby is a teenager now & Cancer Free. Documentary follows 6 terminal cancer patients for 5 yrs, who choose a radical "food" treatment)* | *“This is presented as a personal story and not a medical claim. It doesn’t meet the criteria for misinformation.”* | The tweet discusses misinformation and is intended to promote natural remedies that are more effective in curing cancer than medical treatment. They feature a supplement link in bio, as such have an interest in promoting such supplements/ alternate cures |
| 3 | Identifies as a person who uses natural  therapy | Surprised reaction to a pregnancy symptom (sweating blue).  (*Doctors Reveal Why Sweat Surprisingly Turns Blue During Pregnancy.* ) | *“The post is framed as a question or reaction, not a claim. It doesn’t constitute misinformation.”* | Tweet discusses false information and contains quotes or references from unrecognised experts |

[3] Ethics Approval:


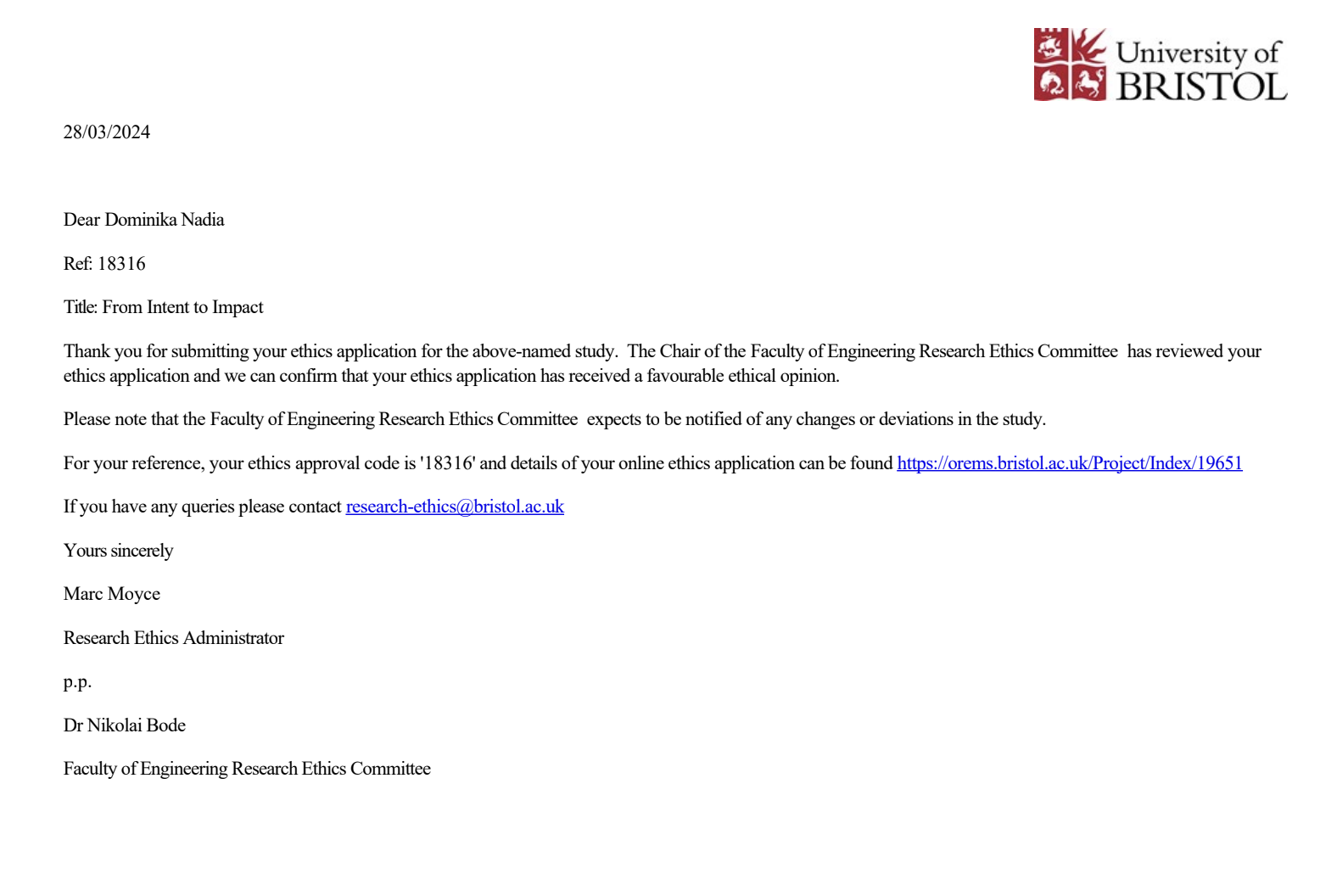

Supplement: Multimedia Appendix 1 [file infodemiology_v6i1e72524_app1.docx]
